# Reconstruction with a novel combined hemipelvic endoprosthesis after resection of periacetabular tumors involving the sacroiliac joint: a report of 25 consecutive cases

**DOI:** 10.1186/s12885-019-6049-7

**Published:** 2019-08-30

**Authors:** Bo Wang, Changye Zou, Xiaokun Hu, Jian Tu, Hao Yao, Junqiang Yin, Gang Huang, Xianbiao Xie, Jingnan Shen

**Affiliations:** 1 Department of Musculoskeletal Oncology, the First Affiliated Hospital of Sun Yat-Sen University, 58 Zhongshan Second Road, Guangzhou, Guangdong China; 2 Reproductive Medicine Center, the First Affiliated Hospital of Sun Yat-Sen University, 58 Zhongshan Second Road, Guangzhou, Guangdong China

**Keywords:** Hemipelvic endoprosthesis, Pelvic tumor, Reconstruction, Limb salvage

## Abstract

**Background:**

Our purpose was to examine the outcomes of patients who underwent extensive resection of periacetabular tumors involving the sacroiliac joint and joint reconstruction with a hemipelvic endoprosthesis.

**Methods:**

The records of 25 consecutive patients diagnosed with Enneking type I/II/IV pelvic tumors from 2010 to 2016 who received resection and hemipelvic endoprosthesis reconstruction were retrospectively reviewed.

**Results:**

The median follow-up period was 48 months. At the most recent follow-up, 11 patients were alive, with estimated 3- and 5-year survival rates of 45.6 and 38.0%, respectively. Fourteen patients died, with a mean survival of 20.8 months, and 8 patients had local recurrence at an average of 9.3 months after surgery. Distal metastases were detected in 11 patients at an average of 11.0 months after surgery. The total complication rate was 56.0%, and the most common complications were wound healing disturbances (28.0%) and deep infections (16.0%). The prosthesis-related complication rate was 24.0%; periprosthetic infections and aseptic loosening were most common. The estimated 1- and 3-year prosthesis survival rates were 81.2 and 63.2%, respectively. The mean Musculoskeletal Tumor Society score was 48.0%. Function and prosthesis-related complications did not differ significantly after adding an extra screw fixation to the first sacral vertebra.

**Conclusions:**

Reconstruction with the hemipelvic endoprosthesis described herein provides satisfactory function with a relatively low complication rate. Adding an extra screw fixation to the first sacral vertebra was not associated with any improvement in the clinical results after short-term follow-up. Improvement and further studies of this endoprosthesis are needed.

## Background

Malignant pelvic tumors are associated with a poor survival rate. Treatment of patients with extensive malignant tumors is challenging due to the difficulty of reconstruction of pelvic bone defects after resection of tumors with wide involvement. Treatment is particularly difficult when the acetabulum and/or the sacroiliac joint are involved. Whenever possible, limb salvage surgery is performed as this is much more acceptable to patients as compared to hemipelvectomy [1–4]. However, when en bloc resection of the sacral wing is required fixation and stabilization of a prosthesis is challenging [5, 6]. Various techniques have been developed in an attempt to address this issue, but none have provided completely acceptable results. For example, Ji et al. [7] harvested bone from the ipsilateral femoral head, shaped it, and used screws to fix it to the residual sacrum. However, postoperative limb function was unacceptabley poor.

A novel prosthetic reconstruction system using an integrative hemipelvic endoprosthesis that spares the sacrum and is cross-fixed to the ipsilateral pedicles of the fifth (L5) and fourth (L4) lumbar vertebrae was developed. d found them to be very promising. Based on those results, improvements were made to the endoprosthesis. Thus, the preliminary experience and clinical effectiveness of this novel prosthesis need to be investigated and summarized, and improvements made in the prosthetic need to be tested and validated.

Thus, the purpose of this study was to report the outcomes of patients with widely invasive Enneking type I/II/IV pelvic tumors who received surgical resection and reconstruction with the improved hemipelvic endoprosthesis at our center over the past 6 years.

## Methods

### Patients

The records of 25 consecutive patients (17 males and 8 females; average age, 24 years; range, 14 to 58 years) who underwent Enneking type I/II/IV resection and pelvic reconstruction at our center from 2010 to 2016 were retrospectively reviewed. All patients received reconstruction with the combined hemipelvic endoprosthesis. Patient characteristics are summarized in Table 1. Written informed consent was obtained from all patients, or from their parents if they were younger than 18 years old. This study was approved by the Ethics Committee of the First Affiliated Hospital of Sun Yat-Sen University.

**Table 1:** Detailed characteristics of the 25 patients

Pathological diagnosis	No.	Wide resection	Marginal resection	Intralesional resection	Major complications^a^ (%)				
					WP	DI	AL	DL	BK
Osteosarcoma	10	5	2	3	2	1	1	1	0
Ewing’s sarcoma	9	4	2	3	3	2	0	0	0
Chondrosarcoma	4	2	1	1	2	1	0	0	1
Metastasis	2	1	1	0	0	0	1	0	0
Total	25	12	6	7	7	4	2	1	1

^a^ *WP* Wound Problem, *DI* Deep Infection, *AL* Aseptic Loosening, *DL* Dislocation, *BK* Breakage

The indications for surgery were the same as previously reported [7]. In brief, indications were: 1) primary or metastatic malignancy; 2) solitary metastasis of an otherwise well-controlled tumor; 3) adequate response to induction chemotherapy; 4) pre-operative work-up suggested that limb salvage surgery will provide adequate surgical margins; and 5) no involvement of the iliac vessels or sciatic or femoral nerve apparent on imaging studies.

Patients were not eligible for surgery if: 1) extensive invasion was present; 2) response to chemotherapy was poor; 3) expected survival time was < 1 year; 3) there was local tumor contamination from an open biopsy; and 4) judged to not be able to tolerate the surgery.

National Comprehensive Cancer Network (NCCN) guidelines were followed for the administration of neoadjuvant chemotherapy. Osteosarcoma treatment regimens included doxorubicin, cisplatin, methotrexate and ifosfamide. Ewing sarcoma was treated with vincristine, etoposide, doxorubicin and ifosfamide. Except 4 patients with conventional chondrosarcoma, patients with primary sarcomas received 2 cycles of chemotherapy preoperatively, and 4–6 cycles postoperatively. Two patients with Ewing sarcoma received radiotherapy.

#### Endoprosthesis

**Fig. 1:**
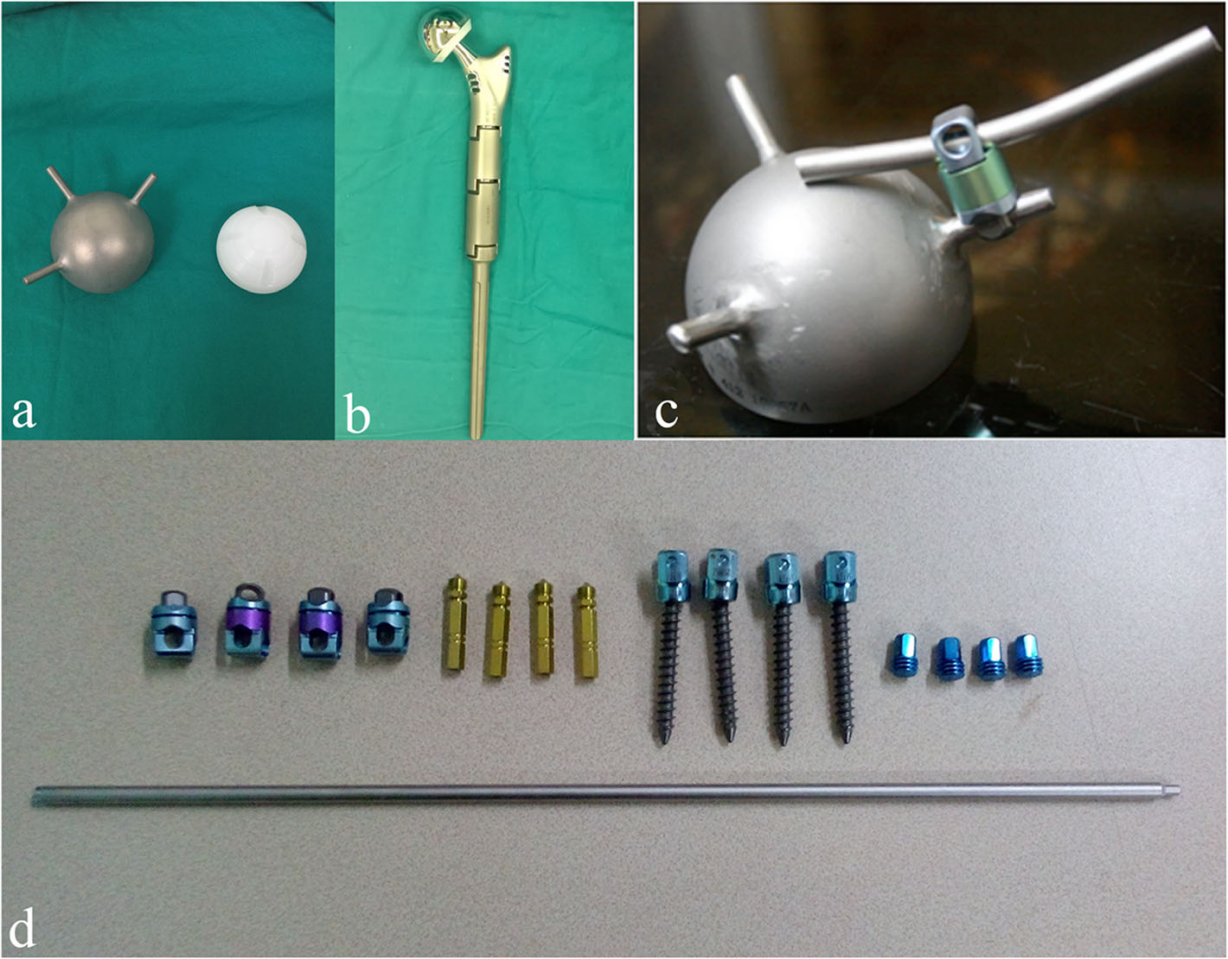
The combined hemipelvic endoprosthesis. **a** A custom-designed acetabular component with 3 connecting rods arranged 120° apart on the top with a polyethylene liner. **b** Cementing of the proximal femoral prosthesis. **c** Connection between the acetabular prosthesis and the pedicle rods. **d** Standard pedicle screw and rod system

**Fig. 2:**
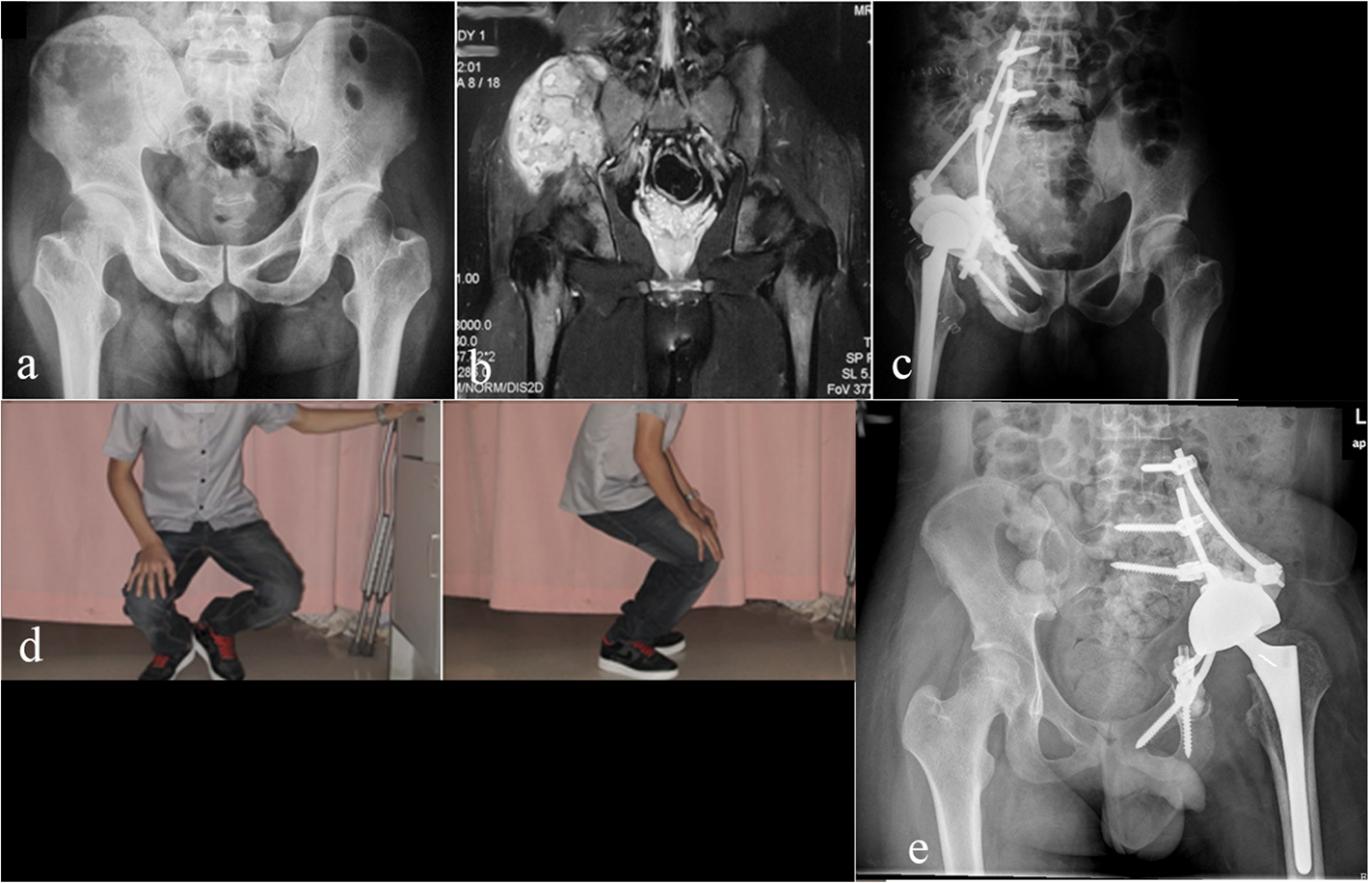
Reconstruction with the combined hemipelvic endoprosthesis after tumor resection. **a** Preoperative X-ray of a patient with osteosarcoma. **b** Preoperative magnetic resonance imaging (MRI) showing involvement of sections I, II and IV of the pelvis. **c** Postoperative X-ray showing reconstruction with the combined hemipelvic endoprosthesis. **d** Functional status 11 months after surgery. **e** Extra screw fixation to the S1 vertebra added based on finite element (FE) study results

The prosthesis system used was described in a prior report [7]. Briefly, the endoprosthesis is a custom-designed acetabular component with 3 connecting rods on the top. The rods are positioned at an angle of 120° to each other. A standard cemented proximal femoral prosthesis and a standard pedicle screw and rod system are also used (Medtronic; Minnesota, USA) (Fig. 1). The acetabular component of the endoprosthesis is crosslinked and fixed with pedicle rods and screws to the ipsilateral pedicles of the fourth (L4) and fifth (L5) lumbar vertebrae. Based on the results of a prior biomechanical study [7], an extra screw fixation to the first sacral (S1) vertebra was added to improve prosthesis fixation (Fig. 2).

#### Surgical procedure

The surgical procedure has been described previously [1]. The procedure consists of a combined extended ilioinguinal and Smith-Petersen approach. In order to achieve large surgical margins, the gluteus medius and minimus were resected when necessary. Ligation of the ipsilateral internal iliac artery was often performed for control of bleeding. In all cases, microwave ablation was used to help reduce contamination by tumor cells and reduce bleeding.

The Enneking and Dunham’s classification system [8] was used to categorize surgical margins (Table 1). For primary tumors, our protocol required wide margins. For metastases, marginal and intralesional resections were both considered acceptable.

### Postoperative management

All patients received intravenous antibiotics (a second generation cephalosporin) postoperative for prophylaxis against infection. The duration of administration was 2 weeks. Sequential compression devices to the lower legs were applied to prevent deep vein thrombosis, and anticoagulants were added if indicated by Caprini scale. Patients were kept at bed rest for approximately 8 weeks, and during this time the hip joint was restricted to mild abduction and external rotation. After 8 weeks, patients began ambulation and progressive weight bearing.

### Follow-up

Patients were seen every 3 months at the outpatient clinic for the first 2 years after surgery. For the next 3 years, they were seen twice a year, and thereafter yearly. Follow-up visits included physical examination, assessment of function, and radiographic studies. Functional assessments were made using the Musculoskeletal Tumor Society (MSTS) scale [9].

### Statistical analysis

Estimates and comparisons of overall survival, local recurrence, distal metastasis, and prosthesis survival were performed using Kaplan-Meier survival analysis. Rates between 2 groups were compared with the log-rank test. A value of *p* < 0.05 was considered to indicate statistical significance. All statistical analyses were performed using the Statistical Package for the Social Science (SPSS) software, version 19.0 (SPSS Inc., Chicago, IL, USA).

## Results

The median follow-up period of the 25 patients included in the study was 48 months (range, 23–87 months). The resection types and surgical margins are described in Table 1.

### Oncological outcomes

**Fig. 3:**
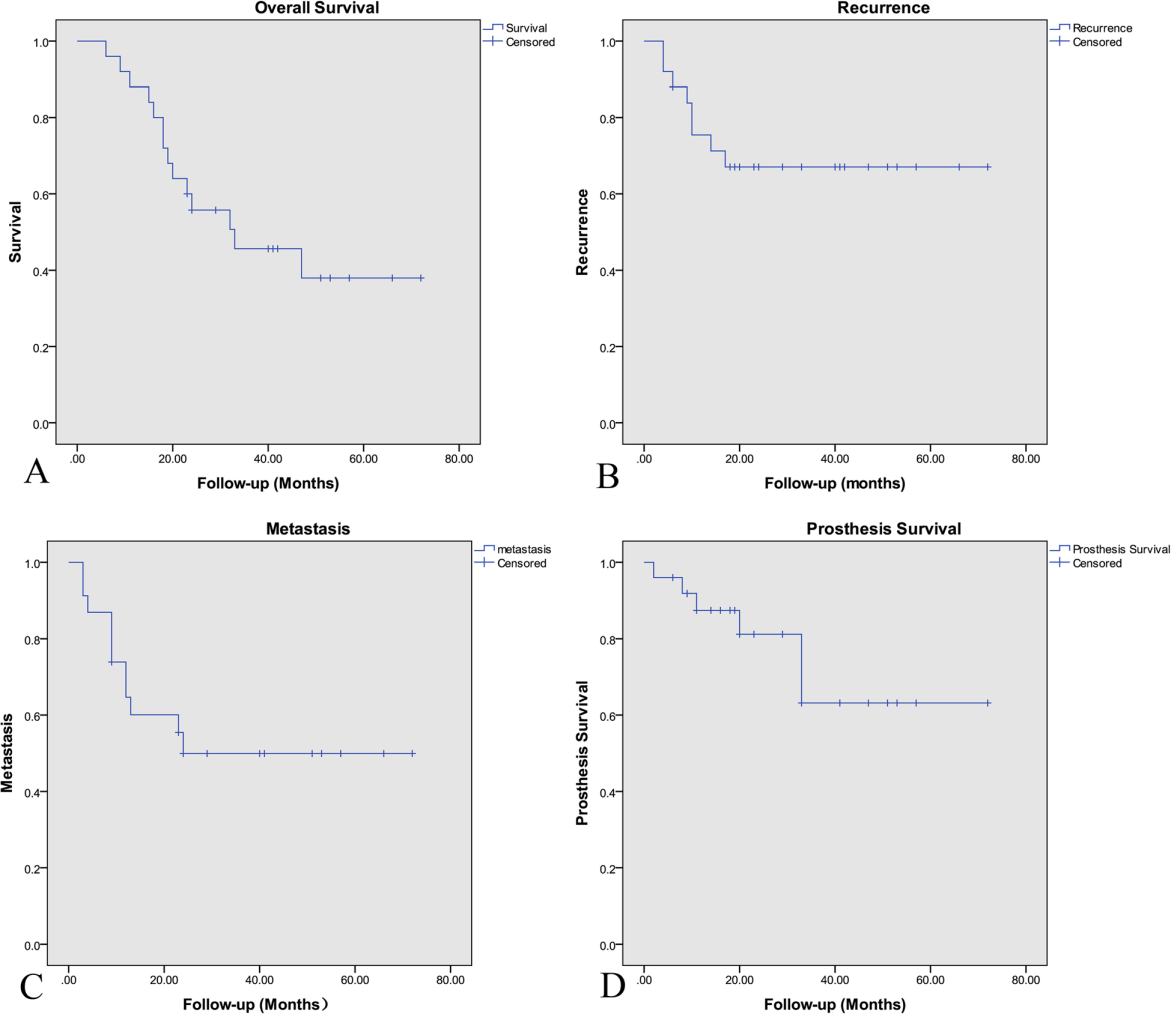
Kaplan-Meier survival analysis of the 25 patients. **a** Overall survival. **b** Disease recurrence. **c** Disease metastasis. **d** Prosthesis survival

Of the 25 patients, 11 were alive at the last follow-up. The estimated 3- and 5-year survival rates were 45.6 and 38.0%, respectively (Fig. 3). Fourteen patients (13 with primary tumors, 1 with metastases) died an average of 21 months after surgery. Eight patients experienced local recurrence; the average disease-free interval was 9 months (range, 4–17 months). The estimated 1- and 3-year recurrence rates were 24.6 and 33.0%, respectively. Of the 8 patients with local recurrence, the surgical margins were wide in 1 patient, marginal in 3 patients, and 4 patients had intralesional margins. Two of the patients with recurrence had Ewing’s sarcoma ad received radiotherapy, 1 patient with osteosarcoma underwent a second limb salvage surgery, and 3 patients underwent hemipelvectomy. The remaining 2 patients declined further treatment.

Distant metastases occurred in 11 patients at an average of 11 months after surgery (range, 3–24 months). These patients all died of their disease an average of 19.3 months after surgery. The estimated 1- and 3-year metastasis rates were 26.1 and 50.0%, respectively.

One of the 2 patients who received surgery for a metastatic malignancy was alive at the most recent follow-up. The other experienced disease progression without local recurrence, and died 47 months after surgery. The survival rates (*p* = 0.57) and recurrence rates (*p* = 0.36) of patients who received surgery for a metastatic malignancy were not different from those who received surgery for a primary tumor.

### Perioperative complications

The operations took an average of 8.7 h (range, 6–22 h), and the mean blood loss was 5600 ml (range, 800–17,000 ml). Fifteen patients (60.0%) were hemodynamically unstable during surgery, and required admission to the surgical intensive care unit (SICU) postoperatively. The average length of SICU stay was 4 days (range 1–9 days).

Problems of wound healing occurred in 7 patients (28%). Three patients experienced fat necrosis, 2 experienced skin necrosis, and superficial wound infections occurred in 2 patients. Three of the patients required a total of 8 debridement surgeries. Four patients (16.0%) developed deep infections, which typically presented a mild fever, continuous discharge, or fistula formation. These patients were treated with intravenous antibiotics, and received a total of 9 debridement surgeries. Two patients required prosthesis removal without further reconstruction.

In addition to the aforementioned complications, there were 2 cases of sciatic nerve injury, 1 case of ureteral injury that required a nephrostomy and a 2-stage repair surgery, and 2 cases of deep vein thromboses (DVT), neither of which led to the development of pulmonary embolism. All patients recovered after conservative therapy.

### Prosthesis-related complications

The overall prosthesis-related complication rate was 24.0%. There were 2 cases of aseptic loosening of the pedicle screw, 1 case of hip dislocation, and 1 case of breakage of the pedicle rod. Two patients developed periprosthetic infections, and required prosthesis removal (Table 1). All patients with prosthesis-related complications required revision surgeries; thus, the overall explantation rate was 24%. The estimated 1- and 3-year prosthesis survival rates were 81.2 and 63.2%, respectively (Fig. 3).

As previously indicated, an extra screw fixation was added to S1 to improve the stability of the prosthesis in some patients based on a prior study [7]. In the present study, 5 patients received extra S1 screw fixation. No significant difference was observed in prosthesis-related complication rates between these 5 patients and the other patients (*p* = 0.83).

### Functional status

The mean MSTS score was 48.0% (range, 30.0–66.7%). The categories of pain reduction and emotional acceptance had the highest overall scores. Restricted lower limb function and limited walking ability was present in all patients, and a brace or a crutch was required for ambulation (Fig. 2). The MSTS scores of patients with extra S1 screw fixation did not differ significantly from those of the other patients (*p* = 0.64).

## Discussion

Limb salvage surgery combined with chemotherapy and radiotherapy for the treatment of pelvic tumors is associated with similar survival and recurrence rates as traditional hemipelvectomy [9–11]. However, limb salvage surgery for periacetabular tumors with wide invasion into the sacroiliac joint (Enneking type I/II/IV) is relatively contraindicated due to difficulties of prosthesis fixation and stabilization after resection of the sacral wing. To address this problem, we designed a hemipelvic endoprosthesis that transfers body weight to the lower limb through the lumbar vertebrae. As such, the sacrum is not required for fixation, which facilitates en bloc resection of tumors.

### Oncological outcomes

In this study, the estimated 3- and 5-year overall survival rates were 45.6 and 38.0%, respectively. These results are certainly not comparable to those of Enneking type I/II or Enneking type I/II/III resections [5, 12, 13]. There are considerable difficulties associated with wide en bloc resection and local control of malignancies invading the acetabulum and sacroiliac joint. These tumors can penetrate the cartilage layer of the sacroiliac joint; therefore, the late recurrence and metastasis rates are increased leading to poor outcomes. However, surgical resection and adjuvant treatment can achieve a clinical cure rate of around 38.0%. In our study, patients with selected metastatic malignancies that were thought to be associated with a poor life expectancy had survival and recurrence rates that were comparable to those of patients with primary sarcomas. This result is in accordance with those of other studies [5, 12]. This suggests that patient selection criteria are important; this procedure is most suitable for patients in whom the primary lesion is controlled and has a single metastasis.

The estimated 1- and 3-year recurrence rates in this study were 24.6 and 33.0%, respectively. These are higher than those reported for Enneking type I/II or Enneking type I/II/III resections. The recurrence rates in patients with metastatic malignancies were not different from those with primary malignancies. Surgical margins that are not clear are believed to be the most important risk factor for recurrence [13, 14]. We found a significant relation between surgical margins and recurrence rates, which is consistent with the results of a prior study [7]. It is difficult to achieve wide and en bloc resection for Enneking type I/II/IV tumors. In this situation, excision with the widest margins possible should be performed, and in the event that only marginal margins can be achieved microwave ablation should performed to reduce the risk of recurrence. Microwave ablation also has the advantages of preventing tumor contamination and reducing bleeding.

### Functional outcomes

Although MSTS scores were relatively lower than those of patients who receive Enneking type I/II or I/II/III resections, we consider the functional results in the current study acceptable because the overall extents of the resections were more extensive. Most of our patients were able to perform activities of daily living. The MSTS scores of patients with and without an extra S1 screw fixation were not significantly different. However, the utility of an extra screw fixation should be investigated in a study with a larger number of patients and longer follow-up.

### Complications

It is reasonable for this major surgery to have a relatively high complication rate. Our overall complication rate was 56.0%, and the most common complications were wound-related and deep infections. Problems with wound healing are not uncommon in patients with overall poor conditions who receive large incisions and extensive soft tissue dissection. Ligation of the internal iliac artery also results in insufficient blood supply to the skin.

Deep infection is the most severe complication of surgeries with large implants. The deep infection rate in the current study (16.0%) was higher than that previously reported for Enneking type I/II or I/II/III resections. Risk factors for deep infections include a long surgical time, poor soft tissue coverage, and relative immunosuppression due to neoadjuvant chemotherapy. These factors also make control of deep infections difficult. We treated deep infections with intravenous antibiotics and debridement. Although prosthesis removal without further reconstruction was required in 2 patients, neither of them required a subsequent hemipelvectomy.

The prosthesis-related complication rate was 24.0%, and aseptic loosening of the pedicle screw and rod was the most common mechanical complication. This complication may have occurred because the crosslinked pedicle rods were fixed to screws in L4 and L5. In our previous biomechanical study, stress was concentrated on the feet of the connecting rods of the acetabulum, and on the proximal segment of the pedicle rod and screw [1]. This is likely the underlying mechanism of the high loosening rate of this prosthesis. Adding an extra screw fixation to the S1 vertebra reduces the peak prosthetic stress by 18.3%, and also provides extra support from the anterior column of the spine, thus increasing the stability of the system. However, statistical analysis showed no differences in prosthesis survival and function after adding an extra S1 screw fixation. This finding may be because only 5 patients received the extra screw fixation and it needed to be further observed in long term follow-up of more patients.

The 3-year prosthesis survival rate was 63.2%. Of the patients that required a revision surgery, 33.3% had deep infections. Even with these infections, the prosthesis survival rate should be higher since the incidence of mechanical failure was lower. An advantage of the combine hemipelvic endoprosthesis is that its assembly can be performed in a number of different ways such that compression or damage to key vessels and nerves, the siatic nerve for example, can be avoided. Importantly, we evaluated disease recurrence separate from that of implant failure in order to better compare the results of this technique with other methods.

There are a number of shortcomings of this study. The follow-up period was relatively short for some of the patients. We classified patients by the location of the tumor, and there was heterogeneity with respect to diseases and types of resection. This made comparisons within groups difficult.

## Conclusions

Limb salvage surgery and reconstruction with the unique hemipelvic endoprosthesis is effective for patients with pelvic tumors. Functional outcomes are adequate, and the rates of complications are relatively low. Adding an extra screw for fixation to the S1 vertebra was not associated with any improvement clinical outcomes during a short-term follow-up period.

## Data Availability

All data can be available at request by email to the corresponding author.

## Abbreviations

DVT: Deep vein thromboses
L4: The fourth lumbar vertebrae
L5: The fifth lumbar vertebrae
MSTS: The Musculoskeletal Tumor Society
NCCN: National Comprehensive Cancer Network
S1: The first sacral vertebrae
SICU: Surgical intensive care unit
SPSS: Statistical Package for the Social Science
